# Aspirin Minimized the Pro-Metastasis Effect of Sorafenib and Improved Survival by Up-Regulating *HTATIP2* in Hepatocellular Carcinoma

**DOI:** 10.1371/journal.pone.0065023

**Published:** 2013-05-31

**Authors:** Lu Lu, Hui-Chuan Sun, Wei Zhang, Zong-Tao Chai, Xiao-Dong Zhu, Ling-Qun Kong, Wen-Quan Wang, Ke-Zhi Zhang, Yuan-Yuan Zhang, Qiang-Bo Zhang, Jian-Yang Ao, Jia-Qi Li, Lu Wang, Wei-Zhong Wu, Zhao-You Tang

**Affiliations:** 1 Liver Cancer Institute and Zhongshan Hospital, Fudan University; Key Laboratory for Carcinogenesis and Cancer Invasion, The Chinese Ministry of Education, Shanghai, P.R. China; 2 Department of Hepatobiliary Surgery, Tianjin Medical University Cancer Institute and Hospital (TJCIH); Laboratory of Cancer Prevention and Therapy, Tianjin, Tianjin, P.R. China; 3 Department of Hepatobiliary and Pancreatic Surgery, Cancer Hospital/Cancer Institute, Fudan University, Shanghai, P.R. China; University of Hong Kong, Hong Kong

## Abstract

**Background & Aims:**

We previously demonstrated the pro-metastasis effect of sorafenib in hepatocellular carcinoma (HCC), which is mediated by down-regulation of tumor suppressor *HTATIP2*. The aim of the present study was to determine whether aspirin minimizes this effect and improves survival.

**Methods:**

The effects of sorafenib, aspirin, and combined sorafenib and aspirin were observed in HCCLM3 and HepG2 xenograft nude mice. Tumor growth, intrahepatic metastasis (IHM), lung metastasis, and survival were assessed. Polymerase chain reaction (PCR) array, real-time (RT)-PCR, and Western blotting were used to examine gene expression. The anti-invasion and anti-metastasis effects of aspirin were studied in *HTATIP2*-knockdown and *HTATIP2*-overexpressing HCC cell lines. The molecular mechanism of *HTATIP2* regulation by aspirin was explored.

**Results:**

Aspirin suppressed the pro-invasion and pro-metastasis effects of sorafenib in HCC and up-regulated *HTATIP2* expression. Aspirin did not inhibit the proliferation of HCC cells, but it decreased the invasiveness of HCC with lower expression of *HTATIP2* and increased expression of a set of markers, indicating a mesenchymal-to-epithelial transition in tumor cells. The up-regulation of *HTATPI2* expression by aspirin is most likely mediated through inhibition of cyclooxygenase (COX) 2 expression.

**Conclusions:**

Aspirin minimized the pro-metastasis effect of sorafenib by up-regulating the tumor suppressor *HTATIP2*; this mechanism is mediated through inhibition of COX2.

## Introduction

Hepatocellular carcinoma (HCC) is the sixth-most common cancer and the third-most common cause of cancer death worldwide [1]. Because of late diagnosis, most HCC patients are not candidates for radical therapy [2], [3]. Sorafenib, a tyrosine kinase inhibitor (TKI), has become the first-line therapy for advanced HCC. However, sorafenib prolongs patients’ median survival time by less than 3 months; many patients have to reduce their dosage or discontinue drug therapy because of its adverse effects [4], [5]. Although the literature contains no reports of HCC outcomes after discontinuation of sorafenib, rebound of tumor growth has been reported for other cancers or after discontinuation of other angiogenesis inhibitors [6]–[10]. Preclinical studies demonstrated that, in some situations, anti-angiogenesis drugs may promote metastasis in addition to inhibiting the primary tumor [10], [11]. Our previous study confirmed that sorafenib promotes invasiveness and metastasis of HCC in xenograft models, as indicated by increased intrahepatic metastasis (IHM), lung metastasis, and circulating tumor cells of tumors with higher expression of *HTATIP2* [12]. Because sorafenib is the only approved molecular-target drug for HCC, there is an urgent need for a therapeutic approach that overcomes these adverse effects to improve sorafenib’s efficacy.

Aspirin, a nonsteroidal anti-inflammatory drug (NSAID), is widely used as an antipyretic and analgesic and to treat rheumatism and prevent cardiovascular disease. Recently, its therapeutic and prophylactic effects with regard to malignant tumors have been of interest to us [13]–[19].

In this study, we found that aspirin minimized the pro-invasion and pro-metastasis effects of sorafenib. This synergistic anti-tumor effect can be attributed to up-regulation of *HTATIP2*.

## Materials and Methods

### Cell Culture and Transfection

Six human HCC cell lines were used, including HCCLM3-wt (HCCLM3 without modification, Liver Cancer Institute, Fudan University [20]), HepG2-wt (HepG2 without modification, Shanghai Institute of Cell Biology), and cell lines derived from HCCLM3-wt and HepG2-wt. HCCLM3-LV-shHTATIP2 and HCCLM3-LV-shNon cells were obtained by infecting HCCLM3-wt cells with lentiviral vectors (LVs) encoding shRNA for *HTATIP2* (LV-shHTATIP2) to eliminate its expression; LV-shNon (transfected with a vector) was a control. HepG2-LV-HTATIP2 and HepG2-LV-Non cells were constructed by LV-HTATIP2 or LV-GFP infection of HepG2-wt cells, which were gifts from Guo and Zhao’s laboratory [21], [22]. All the cell lines were cultured in Dulbecco’s Modified Eagle Medium (DMEM) supplemented with 10% fetal bovine serum (FBS).

### Animal Models and Treatments

Male, 6-week-old BALBc nu/nu mice were obtained from the Shanghai Institute of Material Medica, Chinese Academy of Science. All mice were bred in laminar flow cabinets under specific pathogen-free conditions. The experimental protocol was approved by the Shanghai Medical Experimental Animal Care Committee. HCCLM3-wt, HCCLM3-LV-shHTATIP2, HepG2-wt, and HepG2-LV-HTATIP2 cells (1×10^7^) were subcutaneously inoculated into the right flanks of the nude mice. After 3–4 weeks, non-necrotic tumor tissue was cut into 1-mm^3^ pieces and orthotopically implanted into the liver. In the survival observation group, treatment was started 2 weeks after orthotopic implantation of the tumor until they died, and mice were randomly assigned to 4 groups (n = 10 for each group), which received a daily oral dose of vehicle solution (control group), 30 mg/kg sorafenib, 15 mg/kg aspirin, or 30 mg/kg sorafenib and 15 mg/kg aspirin. Another 4 groups (n = 6 mice in each group) received the same dosages, but for 4 weeks, and tumor samples were then extracted for further analysis.

### Drugs and Reagents

Sorafenib (Bayer Healthcare) was prepared as previously described [23], [24]. Briefly, for the in vivo study, sorafenib was formulated at 4 times the highest dose in a Cremophor EL/ethanol (50∶50) solution. This ×4 stock solution was prepared daily. Final dosing solutions were prepared on the day of use by diluting the stock solution to ×1 with endotoxin-free distilled water (Life Technologies) and mixing it by vortexing immediately before dosing. Sorafenib was finally prepared as a ×1 solution with Cremophor EL/ethanol/water (12.5∶12.5∶75, the vehicle solution). For in vitro studies, sorafenib was dissolved in dimethyl sulfoxide, and the final concentration was 5 µmol/L. Aspirin (Sigma; St. Louis, MO), FR122047 (an inhibitor of cyclooxygenase [COX] 1; Tocris Bioscience, Bristol), and NS-398 (an inhibitor of COX2; Sigma; St. Louis, MO) were dissolved in dimethyl sulfoxide for further experiments. The concentration of aspirin used in in vitro studies was 0.1–10 mmol/L. The concentration of FR122047 and NS-398 used in in vitro studies was 30 µmol/L and 50 µmol/L, respectively.

### Detection of Metastasis by Hematoxylin-eosin Staining

Tumors were excised and their largest (*a*) and smallest (*b*) diameters were measured to calculate tumor volume, *V* = *ab* ^2^/2. The excised tumors and lungs were fixed with 4% formaldehyde and embedded in paraffin. Serial sections were cut at 5 µm for histologic study. For each mouse, 30 intermittently selected sections were stained with hematoxylin and eosin and examined for the presence of intrahepatic metastasis (IHM) and lung metastasis. Two pathologists who were blinded to the groups independently evaluated the number of metastatic nodules. The standardized number of lung metastases (SNLM) was increased when the number of lung metastases was standardized by liver tumor size.

### PCR Microarray Analysis of Gene Expression in Tumors

Six tumors from each group were pooled to extract total RNA and were studied using the Human Cancer PathwayFinder RT^2^ Profiler PCR Array, according to the manufacturer’s instructions (http://www.sabiosciences.com/rt_pcr_product/HTML/PAHS-033A.html).

### Quantitative Real-time PCR Analysis

RT-PCR procedures are described elsewhere [23]. The following primers for amplification of human genes were used: *HTATIP2*, forward 5′-TCACCTTCGACGAGGAAGCT-3′, and reverse 5′-GCTCTGCAGACTTCAGACCA-3′; and β-actin, forward 5′-CACCATGAAGATCAAGATCATTGC-3′, and reverse 5′-GGCCGGACTCATCGTACTCCTGC-3′.

### Western Blot Assay

Procedures are described elsewhere [23]. Primary antibodies included anti–E-cadherin (Cell Signaling Technology; Denver, MA), anti–N-cadherin (Abcam; Cambridge, MA), anti-vimentin (Santa Cruz Biotechnology; Santa Cruz, CA), anti-HTATIP2, anti-STAT3, anti-pSTAT3, anti–NF-κB(p65), anti-COX2 (Abcam, Hong Kong), anti-COX1, anti–β-catenin (Epitomics; Burlingame, CA) and anti–β-actin (Kangcheng Technology, Shanghai).

### Immunofluorescence and Immunohistochemistry

To assess the distribution of *HTATIP2* in HCC cells, HCCLM3-wt, HCCLM3-LV-shNon, HCCLM3-LV-shHTATIP2, HepG2-wt, HepG2-LV-Non, and HepG2-LV-HTATIP2 were stained by immunofluorescence. First, cultured cells were grown on slides and then washed and fixed. Cells were then incubated with primary antibody to HTATIP2 (Abcam, Hong Kong) and goat anti-rabbit tetramethyl rhodamine isothiocyanate-conjugated secondary antibody (Santa Cruz Biotechnology; Santa Cruz, CA) before staining with 4′, 6-diamidino-2-phenylindole (DAPI). The fluorescent images were visualized using a confocal microscope (FV-1000; Olympus). The tumor sections were incubated with primary antibody to HTATIP2 (Abcam, Hong Kong), and goat anti-rabbit IgG/horseradish peroxidase (Santa Cruz Biotechnology;, Santa Cruz, CA) was applied as the secondary antibody. For negative controls, primary antibodies were replaced with phosphate-buffered saline. Staining was independently evaluated by 2 observers.

### Cell Proliferation and Invasion Assay

Cell proliferation was counted with a CCK-8 assay (Dojindo; Tokyo, Japan). HCCLM3-wt, HCCLM3-LV-shNon, HCCLM3-LV-shHTATIP2, HepG2-wt, HepG2-LV-Non, and HepG2-LV-HTATIP2 cells were incubated in 96-well plates (5×10^3^ cells/well) with or without aspirin for 48 hours. The procedure for cell proliferation is described elsewhere [23]. For cell invasion assay, transwell chamber inserts (Corning Inc; Corning, NY) with a filter membrane pore size of 8 µm were coated with 80 µL Matrigel (0.8 mg/mL, BD Bioscience). HCCLM3-wt, HCCLM3-LV-shHTATIP2, HepG2-wt, and HepG2-LV-HTATIP2 cells were seeded at a concentration of 5×10^5^/mL in serum-free DMEM and incubated with 0.1 mmol/L and 0.5 mmol/L aspirin or vehicle at the upper chamber. DMEM containing 10% FBS was added to the lower compartment. Seventy-two hours later, cells that had migrated through the permeable membrane were fixed in paraformaldehyde, stained with Giemsa, and counted under an inverted light microscope at ×100 magnification. Each assay was done in triplicate.

### Statistical Analysis

Continuous data were expressed as mean ± SD and were compared with unpaired, 2-tailed Student *t* test or Mann-Whitney *U* test, unless otherwise specified, using SPSS for Windows, version 12.0 (SPSS, Inc). *P*<0.05 (2 sided) was considered statically significant.

## Results

### 1. Aspirin Suppressed the Pro-invasion and Pro-metastasis Effects of Sorafenib in HCC

**Figure 1:**
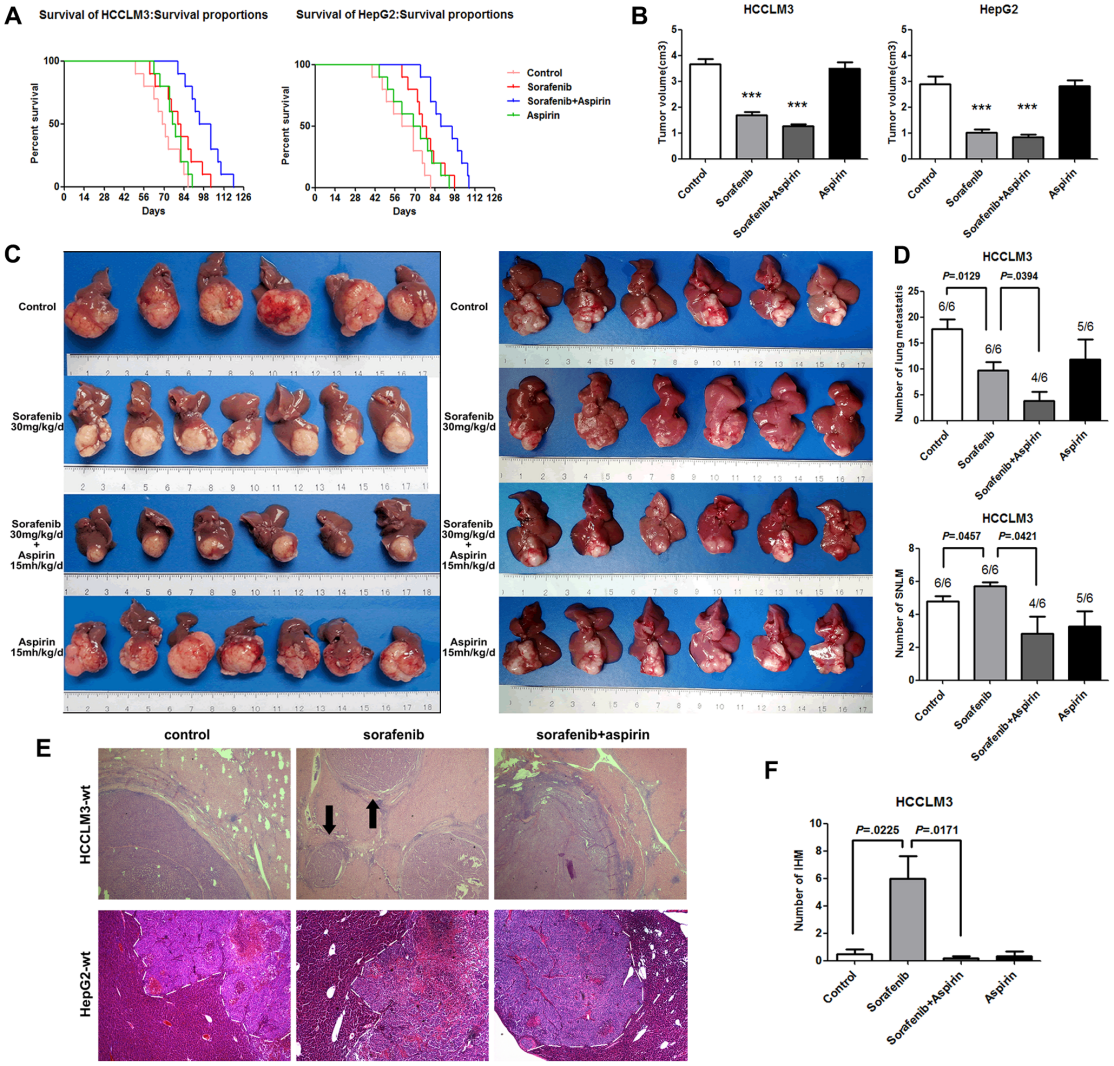
Aspirin suppressed the pro-invasion and pro-metastasis effects of sorafenib in orthotopic HCC models. (A) Median survival was improved by sorafenib treatment with or without aspirin in orthotopic HCCLM3 and HepG2 models. Moreover, sorafenib combined with aspirin significantly prolonged median survival compared to sorafenib alone (98.5±3.85 days vs 81.0±4.31 days in the HCCLM3-wt model, *P = *0.0076; 92.3±3.82 days vs 77.7±3.54 days in the HepG2-wt model, *P = *0.0122). (B) In the HCCLM3-wt model (left panel), tumor size was 1.70±0.12 cm^3^ in the sorafenib group (*P*<0.001), 1.27±0.08 cm^3^ in the group treated with both sorafenib and aspirin (*P*<0.001), and 3.68±0.18 cm^3^ in the control group. In the HepG2-wt model (right panel), tumors were smaller in the sorafenib group (1.02±0.11 cm^3^, *P*<0.001), and the group treated with combined sorafenib and aspirin (0.84±0.11 cm^3^, *P*<0.001), compared with controls (2.90±0.29 cm^3^). (C) In the HCCLM3-wt model (left panel), the sorafenib group had more intrahepatic metastases (IHM), although tumor size was smaller, and the group treated with combined sorafenib and aspirin had significantly fewer IHM. The HepG2-wt model (right panel), was characterized by invasive growth, and sorafenib did not further increase its invasiveness and metastasis. However, tumor margins became clearer in the group treated with combined sorafenib and aspirin. (D) In the HCCLM3-wt model, there were obviously fewer lung metastases in the sorafenib group (9.67±1.71) compared to the control group (17.67±1.94, *P = *0.0129) The incidence of lung metastasis (6/6) was not decreased in the sorafenib group. However, the standardized number of lung metastases (SNLM) was greater in the sorafenib group (5.72±0.24) than in the control group (4.77±0.33, *P = *0.0457). In the group treated with combined aspirin and sorafenib, the incidence of lung metastasis (4/6) and SNLM were distinctly decreased compared with the group treated with sorafenib alone (2.84±1.04 vs 5.72±0.24, *P = *0.0421). (E) Hematoxylin-eosin staining confirmed that sorafenib induced more IHM in the HCCLM3-wt model, and aspirin was able to reverse the adverse effect. In addition, aspirin was able to effect a clearer margin in the HepG2-wt model. (F) Comparison of IHM in the HCCLM3-wt model. The number of IHM was greater in the sorafenib group compared to the control group (6.00±1.65 vs 0.50±0.34, *P = *0.0225) and lower in the sorafenib and aspirin combined therapy group compared to the sorafenib group (0.17±0.17 vs 6.00±1.65, *P = *0.0171).

In both the HCCLM3-wt model and the HepG2-wt model, sorafenib (30 mg·kg^−1^·d^−1^) prolonged survival and decreased tumor volume (Figure 1 A,B). However, in the HCCLM3-wt model, the number of IHM was increased in the sorafenib group (Figure 1 C,E,F), which is in accordance with the previous result. Moreover, although there were fewer lung metastases in the sorafenib-treated mice, the standardized number of lung metastases (SNLM) was greater when the number of lung metastases was standardized by liver tumor size (Figure 1D). In the HepG2-wt model, sorafenib did not promote invasion and metastasis compared with untreated tumors (Figure 1 C,E). When tumors were treated with a combination of aspirin and sorafenib, the median survival was longer compared to that of the sorafenib group, in both the HCCLM3-wt model and the HepG2-wt model (Figure 1A), and the number of IHM was significantly less compared to the sorafenib group in the HCCLM3-wt model (Figure 1C,E,F), and tumor margins were clearer than in the sorafenib group in the HepG2-wt model (Figure 1 C,E). The number of lung metastases, the SNLM, and the incidence of lung metastasis in the HCCLM3-wt model were all lower in the combination treatment group than in the sorafenib group (Figure 1D). However, the aspirin group had neither decreased tumor volume nor prolonged survival compared with control mice (Figure 1 A,B).

### 2. Aspirin Up-regulated Tumor Suppressor Gene *HTATIP2,* which was Down-regulated by Sorafenib

**Figure 2:**
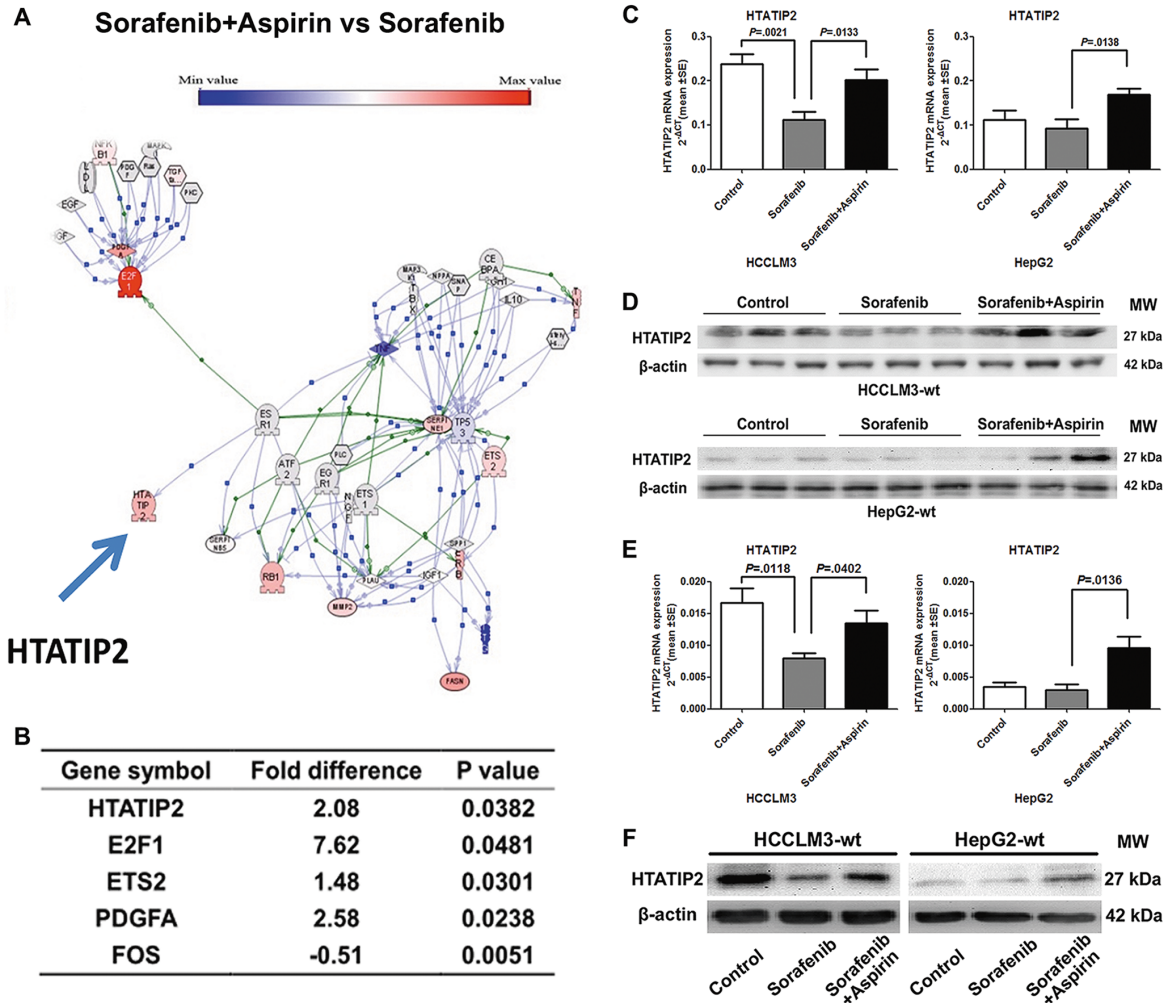
Aspirin up-regulated *HTATIP2,* which was down-regulated by sorafenib. (A) Gene network analysis by Ariadne software shows differences in 84 genes between the sorafenib and aspirin combination group and the sorafenib group. (B) Comparison of gene expression in the sorafenib and aspirin combination group and the sorafenib group. (C, D) Expression of *HTATIP2* in HCCLM3-wt and HepG2-wt tumors was evaluated at the mRNA level (C) and protein level (D). (E, F) Expression of *HTATIP2* in HCCLM3-wt and HepG2-wt cell lines was confirmed at the mRNA level (E) and protein level (F). (Columns, mean of 6 samples in each group; bars, SEM).

We demonstrated in a previous study that sorafenib promotes the invasiveness and metastatic potential of HCC, which is associated with changes in the expression of a number of genes, as shown by a PCR array [12]. We therefore investigated which genes might be responsible for the effect of aspirin. A PCR array profiling the expression of 84 genes representative of the 6 biological pathways involved in transformation and tumorigenesis showed that, in the HCCLM3-wt xenograft model, mice treated with combined sorafenib and aspirin had significantly different expression of 5 genes compared with the sorafenib group (Figure 2 A,B). *HTATIP2*, which was confirmed as the key gene down-regulated by sorafenib to promote the invasiveness and metastatic potential of HCC, was also identified as the key gene up-regulated by combined aspirin and sorafenib. Down-regulation of *HTATIP2* by sorafenib and up-regulation of *HTATIP2* by combination treatment were confirmed by RT-PCR and Western blotting in the HCCLM3-wt model (Figure 2 C,D). Sorafenib did not down-regulate mRNA and protein expression of *HTATIP2* in HepG2-wt tumors compared with untreated tumors, probably because HepG2-wt has low expression of *HTATIP2*. However, combination treatment significantly up-regulated *HTATIP2* expression in the HepG2-wt xenograft model (Figure 2 C,D).

As we showed in the previous study, sorafenib down-regulated *HTATIP2* expression in the HCCLM3-wt cell line, but not the HepG2 cell line. Sorafenib combined with aspirin distinctly up-regulated *HTATIP2* expression when compared with the sorafenib groups of both HCCLM3-wt and HepG2-wt cell lines (Figure 2 E,F).

### 3. Aspirin Inhibited the Invasiveness but not Proliferation of HCC Cells, and Reversed Epithelial-to-mesenchymal Transition by Up-regulating *HTATIP2* Expression

**Figure 3:**
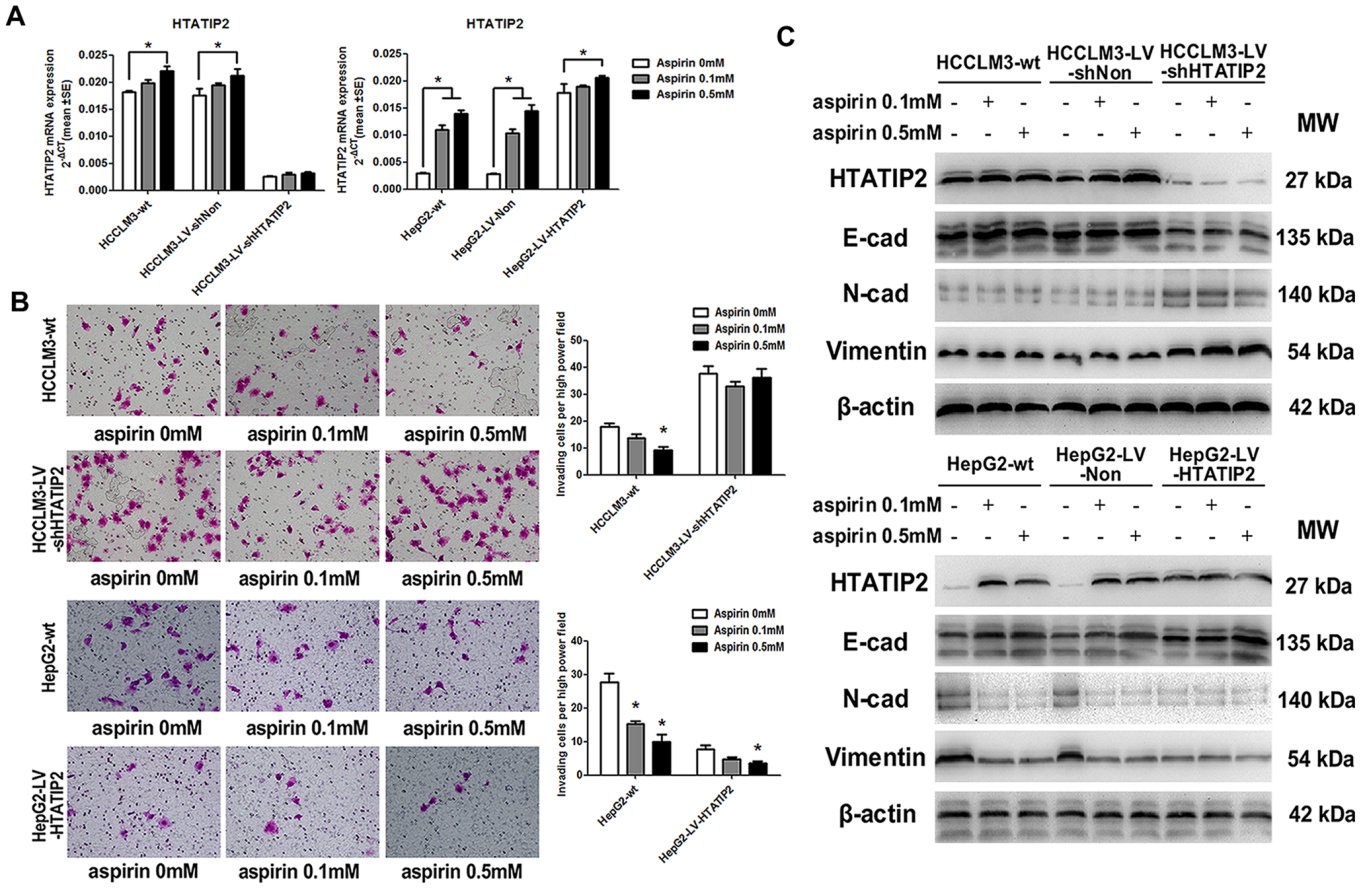
Up-regulation of *HTATIP2* by aspirin reversed epithelial-to-mesenchymal transition (EMT) and inhibited invasion in HCC cell lines. (A) Expression of *HTATIP2* mRNA was detected by RT-PCR (columns, mean of 6 samples in each group; bars, SEM; *, *P*<0.05). (B) Invasion of HCC cell lines with different expression levels of *HTATIP2* was measured by transwell assay. Left panel: migrated tumor cells. Right panel: quantification of invasion assay. (C) Levels of *HTATIP2* protein and EMT markers, including E-cadherin, N-cadherin, and vimentin, were revealed by Western blotting.

HCCLM3-LV-shHTATIP2 (down-regulating *HTATIP2* by siRNA), HCCLM3-LV-shNon (transfected with vector), HepG2-LV-HTATIP2 (up-regulating *HTATIP2*), and HepG2-LV-Non were used to explore the effect of aspirin on *HTATIP2* expression. RT-PCR, Western blotting, and immunofluorescence showed that 0.5 mmol/L aspirin up-regulated *HTATIP2* expression in all HCC cell lines except HCCLM3-LV-shHTATIP2 (Figures 3 A,C and S1).

A 48-hour proliferation assay showed that aspirin had little effect on tumor cells at a concentration of 0.1–1 mmol/L, but it significantly inhibited proliferation at a concentration of 10 mmol/L in HCCLM3-wt and at a concentration of 2–10 mmol/L in HepG2-wt (Figure S2A). Because the concentration of aspirin in human plasma was 0.15–0.3 mmol/L when it was taken at 100–300 mg/d for prevention of cerebro-cardiovascular diseases according to the drug instruction, we used 0.1 and 0.5 mmol/L in the following assays. A 48-hour proliferation assay showed that 0.1–0.5 mmol/L aspirin has no effect on HCCLM3-LV-shNon, HCCLM3-LV-shHTATIP2, HepG2-LV-Non, or HepG2-LV-HTATIP2 (Figure S2B). Cell invasion assay demonstrated that 0.5 mmol/L aspirin inhibited invasiveness of HCCLM3-wt, HepG2-wt, and HepG2-LV-HTATIP2 cells, but not HCCLM3-LV-shHTATIP2 cells (Figure 3B).

*HTATIP2*-knockdown HCCLM3-LV-shHTATIP2 cells had down-regulated expression of E-cadherin and up-regulated expression of N-cadherin and vimentin compared with HCCLM3-wt cells, whereas overexpression of *HTATIP2* in HepG2-LV-HTATIP2 cells changed a panel of epithelial-to-mesenchymal transition markers toward mesenchymal-to-epithelial transition compared with HepG2-wt cells. *HTATIP2* was significantly up-regulated with 0.1–0.5 mmol/L; as a result, E-cadherin was up-regulated and N-cadherin and vimentin were down-regulated in HepG2-wt. However, in HCCLM3-LV-shHTATIP2, aspirin had no effect on expression of *HTATIP2* and epithelial-to-mesenchymal transition markers (Figure 3C).

### 4. Aspirin Up-regulated *HTATIP2* Expression and Suppressed Invasiveness and Metastatic Potential in the HepG2-wt Xenograft Model

**Figure 4:**
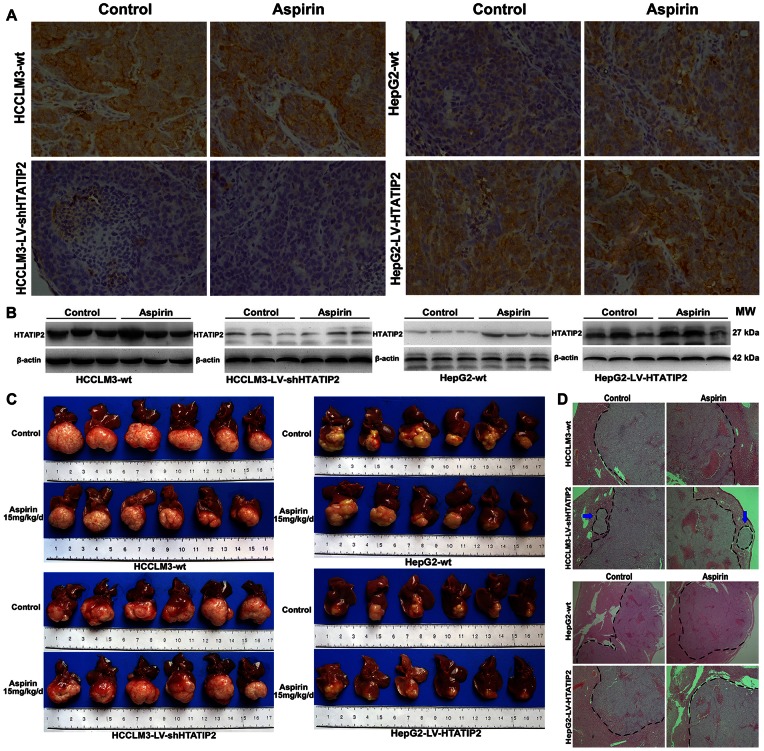
Aspirin up-regulated *HTATIP2* expression and suppressed invasiveness and metastatic potential in a xenograft hepatoma model. (A, B) Expression of *HTATIP2* protein was detected by immunohistochemistry (A, original magnification, ×200) and Western blotting (B). (C) In HCCLM3-wt and HCCLM3-LV-shHTAITP2 models (left panel), *HTATIP2* knockdown apparently increased IHM; aspirin did not affect these 2 models. In HepG2-wt and HepG2-LV-HTATIP2 models (right panel), overexpression of *HTATIP2* obviously inhibited tumor invasion. The tumor margin was clearer with aspirin treatment in the HepG2-wt model. (D) Hematoxylin-eosin staining confirmed that lower expression of *HTATIP2* promoted invasiveness and metastasis potential of HCC, which could be reserved by aspirin.

Immunohistochemistry and Western blotting revealed that aspirin significantly up-regulated *HTATIP2* expression in HepG2-wt tumor tissues, had a mild effect on *HTATIP2* expression in HCCLM3-wt and HepG2-LV-HTATIP2, and had no effect on *HTATIP2* expression in HCCLM3-LV-shHTATIP2 tumor tissues (Figure 4 A,B).

In a xenograft HCC model, aspirin significantly suppressed invasiveness in HepG2-wt cells with low expression of *HTATIP2*. Tumor margins were clearer in aspirin-treated mice than in control mice. However, aspirin had no effect in the *HTATIP2*-knockdown HCCLM3-LV-shHTATIP2 xenograft model; IHM occurred regardless of whether aspirin had been administered (Figure 4 C,D).

### 5. Aspirin Up-regulated *HTATIP2* Expression by Inhibiting COX2 Expression

**Figure 5:**
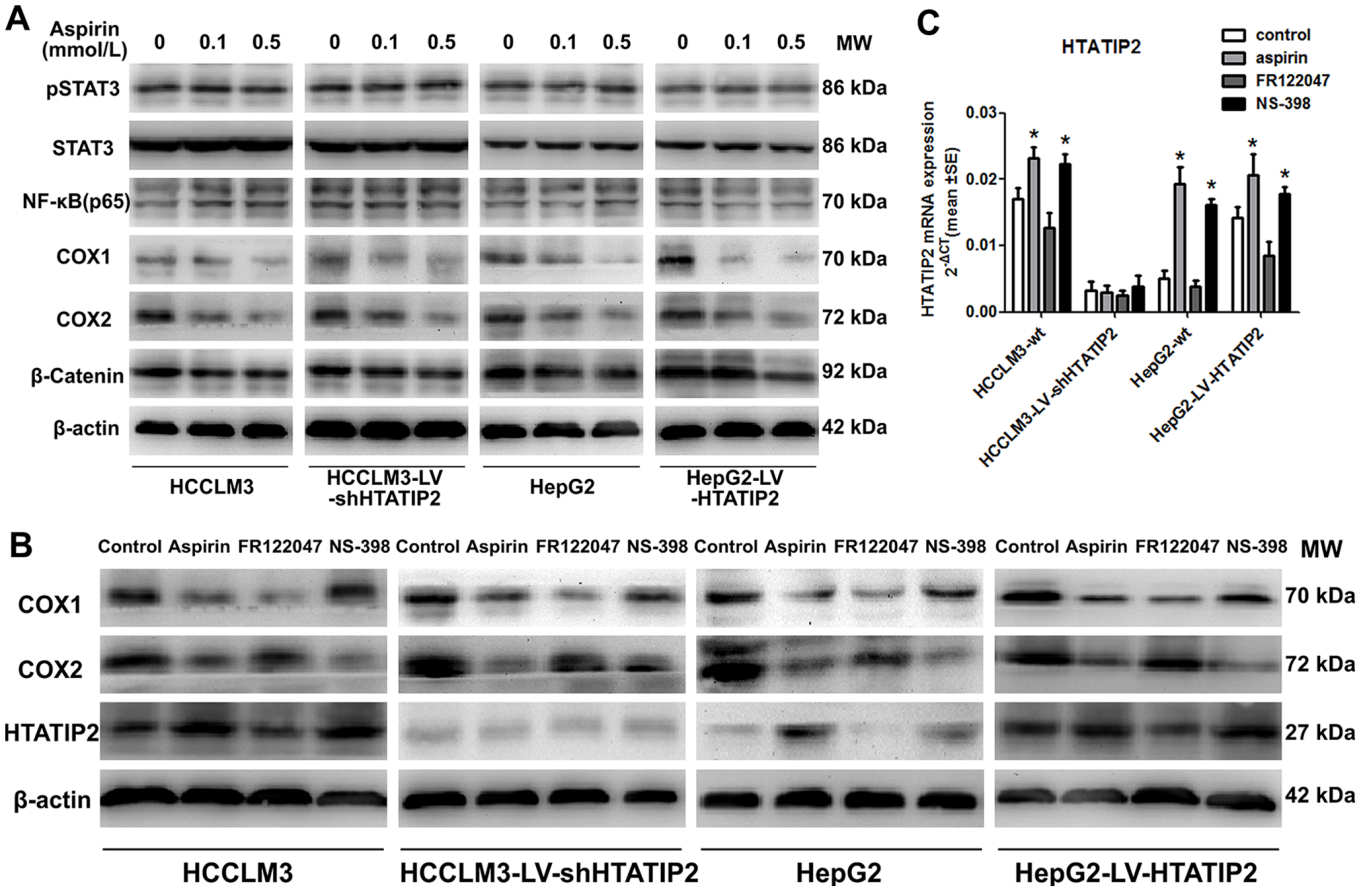
Aspirin up-regulated *HTATIP2* by inhibiting COX2 expression. (A) Western blotting revealed that aspirin down-regulated COX1 and COX2 expression in HCC cell lines. (B) After 12 hours of treatment, Western blotting revealed a down-regulation of COX2 expression by aspirin, and NS-398 corresponded with an increase in *HTATIP2*. (C) The up-regulation of *HTATIP2* mRNA by aspirin and NS-398 was confirmed by real-time PCR (columns, mean of 6 samples in each group; bars, SEM; *, *P*<0.05).

We have reported that *HTATIP2* was down-regulated by sorafenib through the JAK/STAT pathway [12]. In the present study, we detected pSTAT3 and some downstream substrates of aspirin including, NF-κB, COX1, COX2, and β-catenin [25], to study whether they are affected by aspirin. As a result, COX1 and COX2 expression were evidently down-regulated by aspirin in all tested HCC cell lines (Figure 5A). Furthermore, the specific COX2 inhibitor NS-398, but not specific COX1 inhibitor FR122047, had an effect similar to aspirin in up-regulating *HTATIP2* expression in HepG2-wt, but it had no effect in HCCLM3-LV-shHTATIP2 and a mild effect in HCCLM3-wt and HepG2-LV-HTATIP2 (Figure 5 B,C).

## Discussion

The present study demonstrated the synergistic effect of sorafenib and aspirin in prolonging median survival in 2 xenograft HCC models. Furthermore, aspirin suppressed the pro-invasion and pro-metastasis effects of sorafenib in HCC through up-regulation of *HTATIP2*, which was probably mediated by inhibition of COX2 expression.

An increasing number of preclinical studies have revealed that, in certain situations such as dose reduction or discontinuation of treatment, TKIs may promote invasiveness and metastatic potential of tumors despite inhibiting the growth of the tumors [6], [26]–[28]. Potential mechanisms are intratumoral hypoxia [29], induction of other angiogenic cytokines when one is blocked [30], modulation of host environment [10], selection of more invasive and metastatic tumors by blockade of vascular endothelial growth factor pathways [11], and induction of metabolic changes in the tumor [31]. In a previous study we found that low-dose sorafenib promoted invasiveness and metastatic potential of HCC with higher expression of *HTATIP2*, although it inhibited primary tumor growth [12]. Because sorafenib is currently the only drug approved for advanced HCC, there is an urgent need for a therapeutic approach that improves its efficacy by mitigating its pro-tumor effects.

Inflammation has decisive roles in tumor initiation, promotion, invasion, and metastasis [32]. Aspirin, an anti-inflammatory agent [33], has shown usefulness in the prevention and treatment of cancers, especially those in the digestive tract [13]–[15], [18]. The anti-platelet effect of aspirin prevented HCC and improved survival in a mouse model of chronic hepatitis B by reducing the number of intrahepatic HBV-specific CD8^+^ T cells and HBV-nonspecific inflammatory cells and the severity of liver fibrosis [34]. More recently, in a large randomized controlled study, aspirin decreased the incidence of HCC and death from chronic hepatic diseases [35], and another study showed that aspirin was associated with a reduced risk of hepatitis B virus–related HCC recurrence after liver resection [36], which highlights the role of aspirin in HCC. Preclinical studies have also demonstrated the anti-tumor effect of aspirin in HCC. High concentrations (>1 mM) of aspirin inhibited growth of cultured hepatoma cells as well as the synthesis of proteins and nucleic acids [37]. Intratumoral injection of 0.4 mL of 5% aspirin inhibited tumor growth in a rabbit VX2 tumor model [16]. Aspirin inhibited hepatocyte growth factor (HGF)-induced invasiveness of HepG2 cells by inhibiting the kinase activity of extracellular signal–regulated kinase (ERK) 1/2, resulting in the suppression of transcriptional activity of Elk-1 as well as nuclear factor κB (NF-κB) and AP-1 [17]. In chemically induced HCC, aspirin suppressed lung metastasis by down-regulating intercellular adhesion molecule 1 (ICAM-1) and vascular cell adhesion molecule 1 (VCAM-1) or by inhibiting NF-κB signaling [38], [39]. The present study demonstrated that low-dosage aspirin (0.1–0.5 mM) did not inhibit the proliferation of HCC cells; however, it did inhibit the invasiveness of HCC cells with lower expression of *HTATIP2*, which is a known tumor suppressor gene [21], [22], [40]. In the xenograft HepG2-wt model, tumor margins became clearer in the aspirin group than in the control group, but tumor volume did not change in the aspirin-treated HCCLM3-wt and HepG2-wt models. This indicates that aspirin alone may not be effective in inhibiting primary tumor growth in HCC, even in a HepG2-wt model with low expression of *HTATIP2*. This is also supported by the results of the in vitro study, which showed that aspirin did not inhibit proliferation but did inhibit invasiveness in HCC cells with lower expression of *HTATIP2*. The present findings suggest that a combination of sorafenib and aspirin will achieve a better result than either alone, similar to the result of another study wherein aspirin enhanced doxorubicin-induced apoptosis and reduced tumor growth in HCC [41]. The combination of sorafenib and aspirin should be given to patients regardless of *HTATIP2* expression status.

A recent study of ours revealed that sorafenib may down-regulate *HTATIP2* through the JAK/STAT pathway [12]. The present study showed *HTATIP2* can be up-regulated by aspirin, probably through inhibition of COX2 expression, but not pSTAT3, β-catenin, NF-κB, or COX1, although all these factors have been demonstrated as target molecules of aspirin [25]. Other mechanisms that may contribute to the anti-invasion effect of aspirin were not investigated in the present study, eg, inhibition of HGF-induced invasion of HepG2 through ERK1/2 [17]; initiation of cell cycle arrest and apoptosis mediated by increased metabolic and oxidative stress [42]; and inhibition or delay of immune-mediated hepatocarcinogenesis [34].

The main side effect of aspirin in clinical application was gastrointestinal hemorrhage [43], [44]. Patients with cirrhosis often have coagulopathy and thrombocytopenia, and approximately 80% of patients with HCC have cirrhosis. Therefore, caution is warranted when using aspirin for HCC patients. The risk of gastrointestinal bleeding was significantly reduced when the dosage of aspirin was decreased [45]. Pharmacokinetic data showed that the concentration of aspirin in human plasma is 0.15–0.3 mmol/L when patients take a lower dosage (100–300 mg daily) according to the drug instruction. At 0.1 and 0.5 mmol/L, the working concentrations in the in vitro assays were considered low dosages. A dosage of 15 mg/kg aspirin in animals was equivalent to 75–100 mg daily dosage in human beings, when it was converted based on drug mass per body surface area [46]. In addition, aspirin in combination with a histamine-receptor inhibitor or proton-pump inhibitor reduced the risk of gastrointestinal bleeding [47]–[50]. In a large-sample study, aspirin use was associated with reduced risk of death due to chronic liver disease, and regular aspirin use was shown to be safe for patients with chronic liver disease. Therefore, a low dosage of aspirin should be safe for well-selected HCC patients, even those with liver cirrhosis. Furthermore, the present study also suggests that a combination of sorafenib and a COX2 inhibitor should achieve a similar synergistic effect without increasing the risk of bleeding.

In conclusion, we found that aspirin reduced the invasiveness and metastasis of HCC through up-regulation of *HTATIP2* expression, which is mediated by inhibition of COX2 expression. Sorafenib may down-regulate *HTATIP2* expression, resulting in greater invasiveness and metastasis. Therefore, aspirin could be used to improve the efficacy of sorafenib, regardless of *HTATIP2* expression in HCC tumor cells.

## Supporting Information

Figure S1 ***HTATIP2*** **protein expression was revealed by immunofluorescence.** Changes in *HTATIP2* protein level were similar to those detected by Western blotting.
(TIF)

Figure S2 **Proliferation assay for HCC cell lines treated with different concentrations of aspirin.** (A) Proliferation assay was conducted in HCCLM3-wt and HepG2-wt cells treated with different concentrations of aspirin for 48 hours. (B) Proliferation assay was conducted in 6 cell lines treated with aspirin for 48 hours.
(TIF)
