# Supplementary figures and images for: Aspirin Minimized the Pro-Metastasis Effect of Sorafenib and Improved Survival by Up-Regulating HTATIP2 in Hepatocellular Carcinoma
Source: PLoS One. 2013 May 31;8(5):e65023. doi: 10.1371/journal.pone.0065023 (PMC3669011; doi:10.1371/journal.pone.0065023)

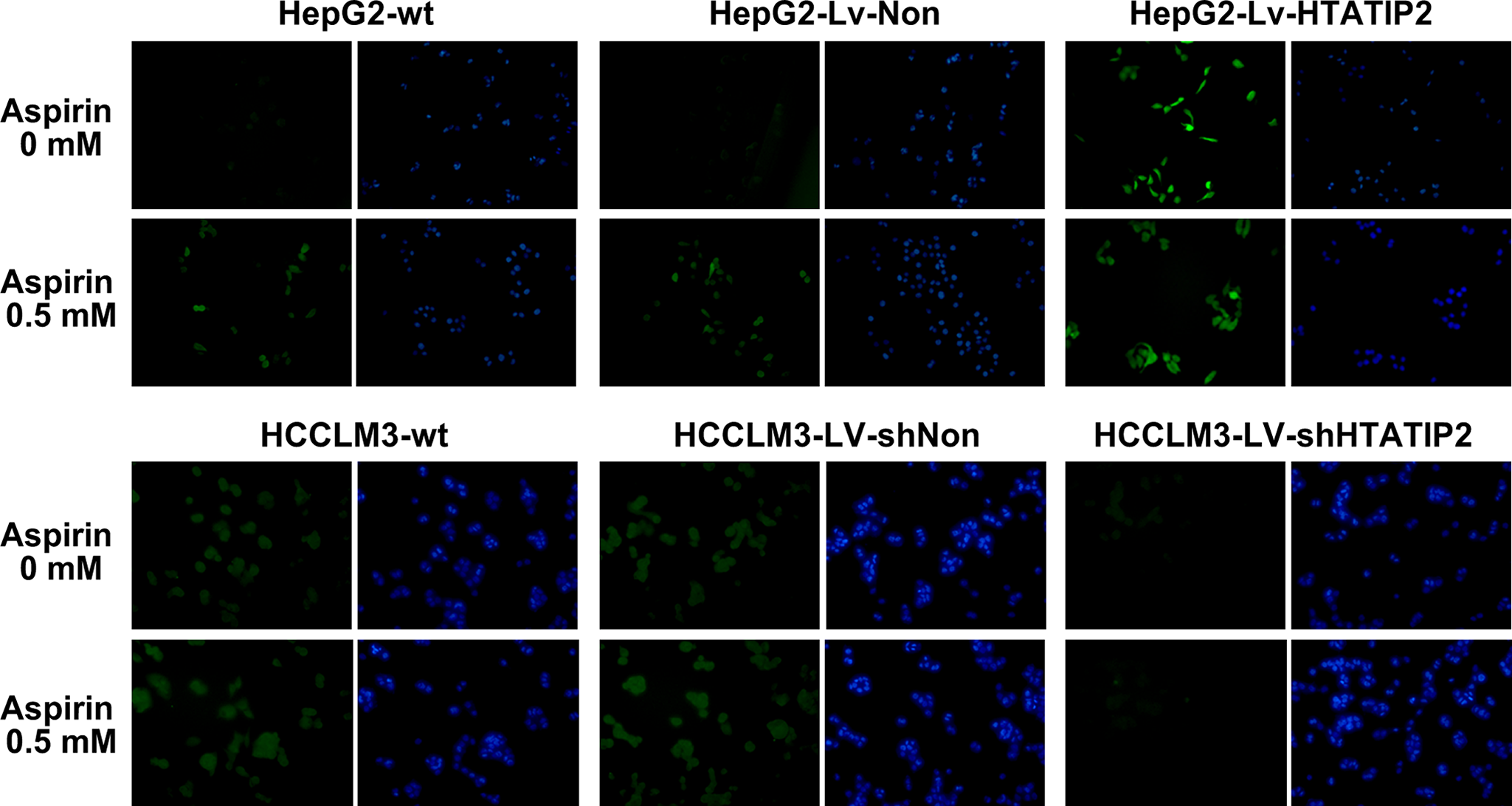

Supplement: Figure S1 — HTATIP2 protein expression was revealed by immunofluorescence. Changes in HTATIP2 protein level were similar to those detected by Western blotting. (TIF) [file pone.0065023.s001.tif]
